# Antisecretory factor in breastmilk is associated with reduced incidence of sepsis in preterm infants

**DOI:** 10.1038/s41390-023-02909-3

**Published:** 2023-11-24

**Authors:** Anna Gustafsson, Ewa Johansson, Ewa Henckel, Axel Olin, Lucie Rodriguez, Petter Brodin, Stefan Lange, Kajsa Bohlin

**Affiliations:** 1 Department of Neonatology, Karolinska University Hospital, SE-17176 Stockholm, Sweden; 2 Department of Clinical Science, Intervention and Technology, Karolinska Institutet, Stockholm, Sweden; 3 Institute of Biomedicine, Department of Infectious Diseases, University of Gothenburg, Gothenburg, Sweden; 4 Sahlgrenska University Hospital, Department of Clinical Microbiology, Västra Götaland Region, Gothenburg, Sweden; 5 Science for Life Laboratory, Department of Women’s and Children’s Health, Karolinska Institutet, and Unit of Infectious Diseases, Karolinska University Hospital, SE-17176 Stockholm, Sweden

## Abstract

**Background:**

Antisecretory Factor (AF) is a protein present in breastmilk that regulates inflammatory processes. We aimed to investigate the level of AF in mothers’ own milk (MOM) in relation to sepsis and other neonatal morbidities in preterm infants.

**Methods:**

Samples of breastmilk and infant plasma were collected at 1, 4, and 12 weeks after birth from 38 mothers and their 49 infants born before 30 weeks gestation. AF-compleasome in MOM was determined by a sandwich enzyme-linked immunosorbent assay (ELISA) and inflammatory markers in infant plasma by a panel of 92 inflammatory proteins. Neonatal treatments and outcomes were recorded.

**Results:**

The level of AF in MOM week 1 was lower for infants with later sepsis compared to no sepsis (*p* = 0.005). Corrected for nutritional intake of MOM, higher levels of AF decreased the risk for sepsis, OR 0.24. AF in MOM week 1 was negatively correlated to inflammatory proteins in infant plasma week 4, markedly IL-8, which was also associated with infant sepsis. Overall, higher AF levels in MOM was associated with fewer major morbidities of prematurity.

**Conclusion:**

Mother’s milk containing high levels of antisecretory factor is associated with reduced risk for sepsis and inflammation in preterm infants.

**Impact:**

- High level of antisecretory factor (AF) in mothers’ own milk is associated with less risk for later sepsis in preterm infants.
- Receiving mothers’ milk with low AF levels during the first week after birth is correlated with more inflammatory proteins in infant’s plasma 2–4 weeks later.
- Human breastmilk has anti-inflammatory properties, and antisecretory factor in mothers’ own milk is a component of potential importance for infants born preterm.
- The findings suggest that food supplementation with AF to mothers of preterm infants to increase AF-levels in breastmilk may be a means to decrease the risk of inflammatory morbidities of prematurity.

## Introduction

Preterm birth (<32 weeks gestational age) may be triggered by inflammation.^1,2^ We have previously reported that cord blood from infants born preterm exhibit a strong pro-inflammatory pattern, with high expression of motif chemokines and interleukin 8, compared to term-born infants.^3^ After birth, preterm infants are exposed to treatments and are at risk for infections that further trigger inflammation. All major morbidities of prematurity, such as bronchopulmonary dysplasia (BPD),^4,5^ necrotizing enterocolitis (NEC),^6^ and retinopathy of prematurity (ROP)^7^ are driven by inflammatory processes. The susceptibility to infections due to immaturity in both barriers and the immune system makes sepsis the most common cause of morbidity and mortality in very low birth weight infants.^8^ Neonatal sepsis has huge implications not only for preterm infants. Globally it is the third leading cause of neonatal mortality and contributes to 13% of all neonatal mortality.^9^ To determine neonatal sepsis, isolation of a pathogen in blood, as well as a combination of clinical symptoms and inflammatory cytokines, is often used.^10^ Very preterm infants with late-onset sepsis show a marked inflammatory response with elevated levels of both pro- and anti-inflammatory cytokines, a pattern that is not yet fully understood.^11^ Despite the development of neonatal care and the availability of antibiotics, sepsis remains a serious condition and may cause long-term consequences.^12^

Breastmilk is bioactive^13,14^ and contains numerous factors that reduce infant mortality, protect against infections, NEC, and other immunological diseases,^15^ and promotes cardiovascular^16,17^ and neurological outcomes.^18^ There is a dose-response relationship for the health effects of breastmilk,^18,19^ but even a limited amount of oropharyngeal administrated colostrum given to preterm infants <32 weeks of gestation has been associated with lower incidence of NEC, severe IVH, and sepsis.^20^ Bioactive factors in breastmilk shape the development of the infant’s innate and adaptive mucosal immune system.^21–23^ There are numerous cytokines and growth factors that are transferred via breastmilk and act as activators.^24^ Non-absorbed human milk oligosaccharides (HMOs) promote gut colonization of bifidobacteria and block the attachment of microbes to the infant’s mucosa, thereby preventing infection.^25^ We have previously shown that lack of bifidobacteria and loss of genes required for HMO utilization is associated with systemic inflammation and immune dysregulation.^24^ There is ample evidence that breastmilk provides a multifactorial anti-inflammatory defence and is the optimal nutrition for all newborn infants,^26^ most likely of particular importance for preterm infants at risk for infections and inflammation.^27,28^

Antisecretory factor (AF) is a protein with a role in the innate regulation of secretory and inflammatory processes,^29^ present in plasma, placenta, and human milk.^29–31^ Normally, AF is present in an inactive form in healthy persons and becomes activated as a part of the innate immune response.^29^ During activation, the AF-including proteasome forms a complex with complement factors, the AF-compleasome, and the AF-compleasome complex act by deactivating the complement activity.^32,33^

Available on the market, there are products to increase levels of active AF, classified as food for special medical purposes (FSMP) by the EU (Commission Directive 1999/21/EC).

Oral intake of specially processed cereals, SPC-Flakes®^29,31,34^ stimulates an increased AF synthesis in both plasma and breast milk. An AF-inducing diet has been reported to decrease symptoms in diseases characterized by inflammation and secretory dysfunction, e.g., inflammatory bowel diseases (IBD), Meniere’s disease,^35–38^ and mastitis.^31^ Preformed active AF can be given directly through oral intake of an egg-yolk solution (Salovum®) with a verified high AF content and has been shown to significantly improve the condition of children with diarrhea.^30,39^

We have described AF in the perinatal period and thus far demonstrated lower levels of AF and more inflammation in placental tissue after preterm pregnancies compared to term.^40^ We have analyzed AF in breastmilk and found that colostrum, which is known to contain high amounts of immune-active components, also have high levels of AF, irrespective of prematurity. AF levels in breastmilk decrease over time but appears to remain higher in milk from mothers of preterm infants, possibly as an adaptive response.^41^ The objective of this study was to focus on infants born preterm and explore if AF levels in mothers’ own milk (MOM) were related to neonatal outcomes, infection, and inflammation.

## Material and methods

### Study population and sample collection

Subjects were recruited within the larger TELLUS study (TELomers, Lung disease and oxidative Stress in preterm infants), a prospective, longitudinal cohort study of cellular aging in preterm infants, between April 2014 and April 2019 at the neonatal intensive care unit (NICU) at Karolinska University Hospital in Sweden. The inclusion criteria for this sub-study cohort were mothers of preterm infants less than 30 weeks gestation that had samples of MOM analysed for AF during the first week post-partum.

Samples of 1–10 ml MOM were collected from mothers who were breastfeeding/expressing milk. Time points for MOM sample collection were at approximately weeks 1, 4 and 12 postpartum. The MOM samples were collected in sterile conical plastic tubes and centrifuged at 2000 g for 10 minutes at room temperature, the supernatant was removed, and the remaining sample was frozen in a −80 °C freezer until analysis.

Plasma samples were collected from infants at similar time points as the MOM samples, approximately 1, 4, and 12 weeks after birth. Whole blood was collected in ethylenediaminetetraacetic acid (EDTA) vials. Plasma was retrieved within two hours after centrifugation twice at 2000 g for 10 minutes and frozen in a −80 °C freezer until analysis.

Maternal and neonatal data were collected prospectively and from medical records. Nutritional data for the infants included the timepoint for the start of enteral nutrition, the day of full enteral nutrition defined as no parenteral nutrition, and days of fasting. The proportion of total nutrition being enteral and the percentage MOM in relation to other enteral nutrition and total nutrition was recorded for each time point for sample collection (weeks 1, 4, and 12 after birth). Neonatal treatments and outcomes were monitored until term corrected age. Sepsis was defined as episodes with clinical symptoms of infection and a positive blood culture.

### Antisecretory factor in breastmilk

For the detection of AF-compleasome (proteasome/complement complexes) in MOM, a sandwich enzyme-linked immunosorbent assay (ELISA) was performed as previously described for plasma^32^ and breastmilk.^41^ Before performing the ELISA, the human milk was pre-treated with ethyl-acetate to remove disturbing fat. Thereafter, monoclonal antibodies (mAbs) against proteasome subunit AF^34^ were used as catching antibodies, and polyclonal antibodies against complement factor C3c were used as detecting antibodies. Samples were assayed at dilution 1/9. Phosphate-buffered saline (PBS) was used as a control. The difference between antibody-coated samples and controls (PBS) was measured, and absorbance was read at 405 nm in a photometer. The expressed net Abs405 nm was used as a proxy for AF-compleasome levels.

### Inflammatory proteins in infant plasma

A panel for multiple plasma protein analysis (ProSeek, Olink AB Uppsala) was used to determine markers of inflammation. The panel included 92 inflammatory proteins, listed in Supplementary Table 1. The method has been previously described.^3,42^ Briefly, paired oligo-nucleotide antibodies with overlapping sequences bind to proteins in the sample. When they are near each other and bound to the target protein, they can be measured using Real-time Polymerase Chain Reaction (PCR). Results from the Olink analyses are demonstrated using Normalized Protein eXpression (NPX). NPX is an arbitrary unit on the log2 scale and allows comparisons across datasets. High levels on the NPX represent high protein concentration.^42,43^

### Statistical analysis

AF-compleasome levels in MOM were analysed in relation to each time point of sampling, in longitudinal series, demographics, and outcome parameters. Spearman’s rho was used to assess the correlation of AF-compleasome between time points and to analyse AF-compleasome related to enteral nutrition parameters. For infant outcomes, the Mann-Whitney *U*-test and the Kruskal Wallis test were used for group comparisons. The Wilcoxon signed rank test and general linear model repeated measures (repeated measures ANOVA) were used for longitudinal samples series. The results of the analysis of inflammatory markers were presented on a log2 scale, therefore non-parametric tests (Spearman’s Rho and Mann-Whitney *U*-test) were used in the analyses. Analyses were performed with the software SPSS (version 27) and with the software R Core Team (Vienna, Austria 2023, https://www.r-project.org/). A *p*-value < 0.05 was regarded as statistically significant.

## Results

### Study group characteristics and levels of AF in MOM

Thirty-eight mothers of preterm infants had samples of MOM analysed for AF during the first week post-partum. There were ten multiple pregnancies (nine twins and one triplet) resulting in 49 infants included for statistical analysis. The gestational age distribution varied between 24^+4^ and 29^+6^ weeks^+days^. The characteristics of the study group are presented in Table 1.

**Table 1:** Maternal and infant characteristics.

Mothers, *n*	38
Infants, *n*	49^a^
**Maternal characteristics**	
Age (years), mean (range)	33 (20–43)
Primiparous, *n* (%)	22 (58)
Multiple pregnancy, *n* (%)	10 (26)
BMI, mean ± SD (min-max)	24 ± 4 (18–35)^b^
GBS urine, *n* (%)	7 (18)
Preeclampsia, *n* (%)	4 (10)
**Mode of delivery**	
Vaginal, *n* (%)	11 (29)
Cesarean section *n*, (%)	27 (71)
ROM > 18 hours, *n* (%)	9 (24)
Pre-/intrapartum antibiotics, *n* (%)	32 (84)
Tocolysis, *n* (%)	23 (60)
Antenatal steroids, *n* (%)	37 (97)
**Infant characteristics**	
Gestational weeks (weeks^days^), mean (range)	27^3^ (24^4^–29^6^)
Female, *n* (%)	24 (49)
Birth weight (grams), mean (range)	1022 ± 239 (458–1623)
Sepsis, *n* (%)	12 (24)
Blood culture verified (days), mean (range)	12 (4–32)

*BMI* Body Mass Index, *GBS* Group B Streptococcus, *ROM* Rupture of Membranes.

^a^Two infants died at postnatal day 10 and 12 respectively.

^b^Missing data, *n* = 1.

A total of 70 samples of MOM were analysed, at week 1 (*n* = 38), week 4 (*n* = 15), and week 12 (*n* = 17) after birth. A week-1 sample was available from all mothers as it was the inclusion criterion for the study. At later timepoints, not all mothers could express milk, and longitudinal analyses were performed in 15 mothers, with samples of MOM at each time point, and their 19 infants (4 sets of twins). Two infants died of NEC at postnatal days 10 and 12 and are therefore only included in the analysis at time point week 1.

Gestational age showed no correlation with AF levels in MOM at week 1 (*n* = 38), week 4 (*n* = 15), or week 12 (*n* = 17) (Spearman’s rho 0.15, *p* = 0.36; 0.38, *p* = 0.16; and 0.003, *p* = 0.99). Infant sex did not affect AF levels in MOM week 1 (Mann-Whitney *U*-test, *p* = 0.84).

Mothers of multiples compared to mothers of singletons exhibited higher AF-compleasome levels at all timepoints (Mann-Whitney *U*-test, *p* = 0.05, 0.003, and 0.04, week 1, 4, and 12, respectively). Other perinatal characteristics, including mode of delivery, parity, rupture of membranes >18 hours, group B streptococci in urine, pre/intrapartum antibiotics, or preeclampsia, were not associated with the AF levels in MOM at any time points (Mann-Whitney *U*-test, data not shown).

### AF in MOM related to infant sepsis

**Fig. 1:**
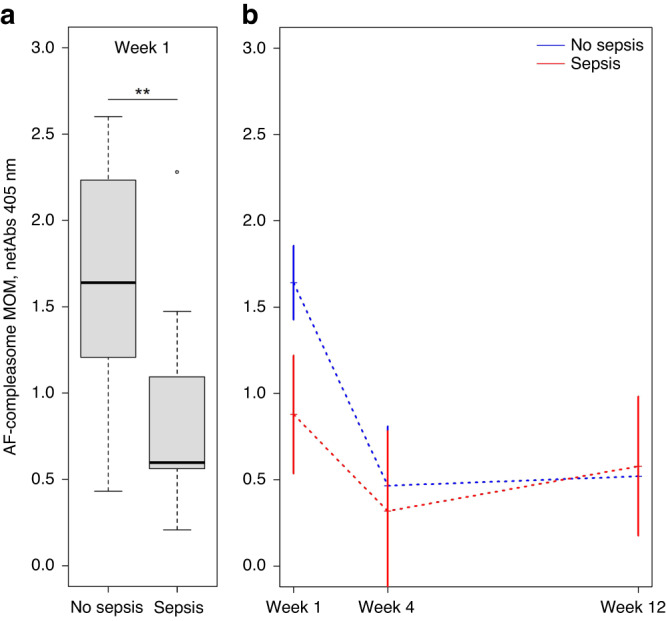
Low antisecretory factor in mothers' own milk is associated to infant sepsis. **a** Detection of AF-compleasome in MOM from mothers after preterm birth using sandwich enzyme-linked immunosorbent assay (ELISA). AF-compleasome in MOM are expressed on the y-axis as mean absorbance values, buffer blank corrected, ± standard deviation (SD). Infant outcome related to sepsis are demonstrated on the x-axis. The boxes represent the 25th to 75th percentiles and the line in the middle represent the median. The whiskers represent the lowest and highest value in the dataset. **p < 0.01. **b** AF-compleasome levels in MOM in longitudinal samples week 1, 4 and 12 (*n* = 19) after birth related to infant sepsis using General linear model, repeated measures, *p* = 0.014. AF-compleasome levels (netAbs) is demonstrated on the Y-axis and sample time point (week 1, 4 and 12) demonstrated on the X-axis. The blue dotted line demonstrates longitudinal values for the group of infants who did not develop sepsis, and the red dotted line infants who developed sepsis.

The levels of AF-compleasome in MOM week 1 were lower for those infants who later developed sepsis versus infants who did not (median 0.70, IQR 0.81 versus 1.73, IQR 1.06, *p* = 0.005, Mann-Whitney *U*-test), shown in Fig. 1a. Using binary logistic regression analysis, higher AF-compleasome levels in MOM week 1 were associated with lower odds of developing infant sepsis (OR 0.24, CI 0.08–0.76, *p* = 0.02). In longitudinal samples, higher AF-compleasome levels were demonstrated in MOM from mothers of infants without sepsis compared to MOM from mothers of infants with sepsis (General linear model *p* = 0.01), shown in Fig. 1b.

Day for the attainment of full enteral nutrition, percentage of enteral nutrition, as well as the percentage of MOM of total nutrition at the time point for sampling may affect absolute AF intake. Infants with sepsis reached full enteral nutrition later than infants without sepsis, median postnatal day 21 (IQR 15) versus 11 (IQR 7), *p* < 0.001 (Mann-Whitney *U*-test).

We estimated if the intake of AF (AF-compleasome level in MOM x proportion of MOM of total nutrition) varied between the groups. These calculations were performed to adjust for differences in both the amounts of enteral nutrition out of total nutrition and the amounts of MOM out of the total enteral intake. AF-compleasome intake in week 1 was lower in infants with sepsis (*n* = 11) versus infants without sepsis (*n* = 31), median 10 (IQR 26) vs 29 (IQR 55), *p* = 0.01 (Mann-Whitney *U*-test), whereas the median percent intake of MOM of total nutrition was not statistically different between the groups, median 17% in the sepsis group vs 23% in the no sepsis group, *p* = 0.12. Of note, if MOM was not reaching the infant’s needs for enteral nutrition, pasteurized donor milk was given, and such milk contains AF with an unknown level.

### AF in MOM related to the number of infant morbidities

**Fig. 2:**
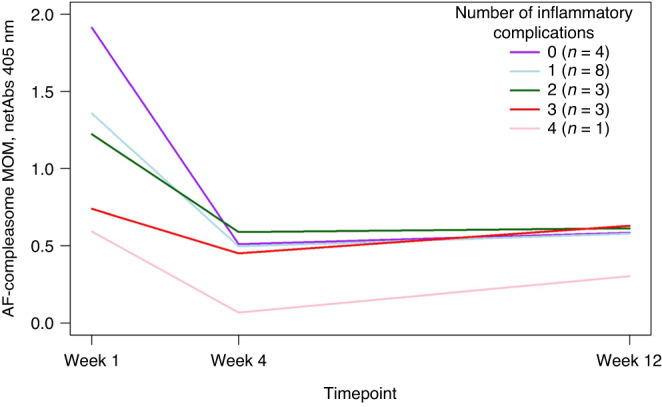
Low antisecretory factor in mothers’ own milk is associated to increasing numbers of major morbidities in the infant. A higher AF-compleasome in MOM week 1 was inversely associated with the number of morbidities (Spearman’s rho −0.361, *p* = 0.01, Kruskal Wallis test *p* = 0.048) (not shown in figure). Longitudinal samples of AF-compleasome in MOM related to number of inflammatory morbidities in the infant (any combination of sepsis, NEC, BPD, PDA, IVH and/or ROP) analyzed using General linear model, repeated measures. AF-compleasome levels (netAbs) is demonstrated on the Y-axis and sample timepoint (week 1, 4 and 12) demonstrated on the X-axis. The different lines demonstrate longitudinal values for the group of infants related to number of morbidities developed.

Several of the infants had more than one adverse outcome. A higher AF-compleasome in MOM week 1 was inversely associated with the number of morbidities (Spearman’s rho −0.361, *p* = 0.01, Kruskal Wallis test *p* = 0.048). Figure 2 visualizes how the number of morbidities relate to levels of AF-compleasome in longitudinal samples (*n* = 15) of MOM (General linear model, *p* = 0.21).

A higher number of infant morbidities were associated with a lower gestational age at birth (Spearman’s rho −0.591, *p* < 0.001, Kruskal Wallis test *p* = 0.002). Infants with more than one major morbidity (specifically infants with sepsis or BPD) had lower gestational age at birth than infants without (data not shown).

### AF in MOM related to the concentration of inflammatory proteins in infant plasma

**Fig. 3:**
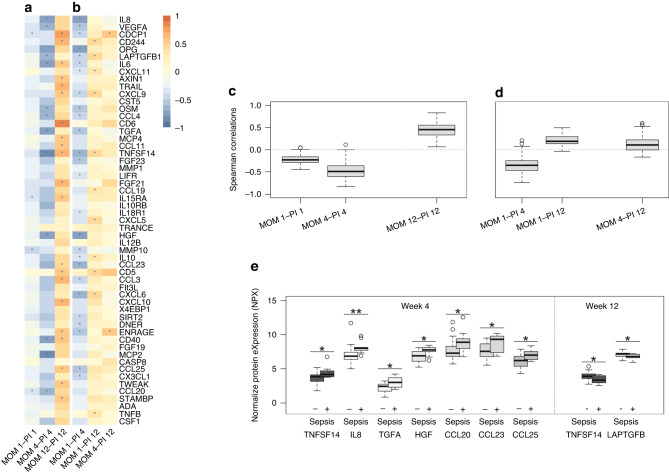
Inflammatory plasma proteins and neonatal sepsis in relation to antisecretory factor in mothers’ own milk. MOM 1, 4 or 12: Antisecretory factor (AF) in mothers’ own milk (MOM) week, 1, 4 or 12 after birth. PI 1, 4 or 12: Protein Infant week 1,4 or 12 after birth. **a** Heat map demonstrating correlations between AF-compleasome levels in MOM and inflammatory proteins in infant plasma at week 1, 4 and 12. Stars (*) illustrate significance level *p* < 0.05. **b** Heat map demonstrating correlations between AF-compleasome levels in MOM week 1 and 4 related to inflammatory proteins in infant plasma at week 4 and 12. Stars (*) illustrate significance level *p* < 0.05. **c** Aggregated protein analysis demonstrating correlations between AF-compleasome in MOM and all inflammatory proteins in the panel at each time point, week 1, 4 and 12. Correlation coefficients are shown on the y-axis and time points on the x-axis. Boxes represent the 25th to 75th percentiles and middle line the median. Whiskers represent the lowest and highest value in the dataset. Single dots represent outliers with distance of 1,5 times over the 75th percentile. **d** As in **c**, demonstrating correlations between AF in MOM week 1 and 4 and proteins in infant plasma later time points, week 4 and 12. **e** Specific inflammatory markers in infant plasma related to sepsis or no sepsis. Results from ProSeek panel of inflammatory markers (Olink) are shown on the y-axis as Normalized Protein eXpression (NPX), an arbitrary unit on the log2 scale where high NPX represent high protein concentration. Group category, infant sepsis (+) or no sepsis (−) at different the different time points is demonstrated on the x-axis. Whiskers represent the lowest and highest value in the dataset. Single dots represent outliers with distance of 1,5 times over the 75th percentile.

The panel of 92 proteins yielded results in our material where several proteins showed a non-negligible proportion of samples with values below the limit of detection (LOD). The limit for exclusion is not fixed and decided on a study-by-study basis. We followed the recommendations of the test manufacturer and chose to exclude proteins with less than 80% of samples above LOD. Thirty proteins were excluded due to LOD, and further, seven were excluded to a low number of samples analyzed, all excluded proteins are marked in Supplementary Table 1. Correlations for all the remaining inflammatory proteins in the panel and AF-compleasome in MOM at the various timepoints are illustrated in heat maps, Fig. 3a, b, and Supplementary Table 2. Correlations were considered weak if the correlation coefficient was less than 0.5.

Overall, combining all proteins analyzed in the inflammatory panel at each time point, the pattern was that AF-compleasome in MOM showed an inverse correlation to most inflammatory proteins in infant plasma at week 1, an even stronger inverse correlation with a wider distribution at week 4, changing with time to a positive correlation at week 12, illustrated in Fig. 3a, c.

Correlations between AF levels in MOM and protein levels in infant plasma at later timepoints demonstrated that AF-compleasome levels in MOM week 1 had a clear negative correlation with most inflammatory proteins in infant plasma in week 4, which was attenuated in week 12 (Fig. 3b, d).

Correlations of 0.5 or more at each timepoint between AF in MOM and proteins in infant plasma are demonstrated in Table 2.

**Table 2:** Correlations of 0.5 or more between antisecretory factor in mother’s own milk (AF MOM) and proteins in infant plasma at different timepoints.

	AF MOM wk 1 - infant plasma wk 4		AF MOM wk 4 - infant plasma wk 4		AF MOM wk 4 - infant plasma wk 12		AF MOM wk 12 - infant plasma wk 12	
**Protein**	*rho*	*N*	*rho*	*N*	*rho*	*N*	*rho*	*N*
IL8	−0.74^Ɨ^	28	−0.75*	11				
VEGFA	−0.54**	28	−0.68*	11				
CDCP1					0.59*	15	0.78**	16
CD244							0.67**	16
OPG	−0.71**	28						
LAPTGFB1			−0.66*	11				
IL6			−0.79*	8			0.59*	13
AXIN1							0.56*	16
TRAIL							0.60*	16
CXCL9							0.55*	16
OSM	−0.56**	28	−0.65*	11				
CCL4	−0.50**	28	−0.67*	11				
CD6							0.83^Ɨ^	16
TGFA	−0.57**	28	−0.71*	11				
MCP4							0.51*	16
CCL11							0.50*	16
TNFSF14	−0.62**	23	−0.80**	11			0.68**	16
FGF23	−0.51*	28						
FGF21							0.61*	13
IL15RA							0.59*	16
HGF	−0.71^Ɨ^	28	−0.78**	11				
CCL23	−0.61**	28						
CD5							0.57*	16
CCL3							0.64**	16
CXCL6	−0.63^Ɨ^	28						
CXCL10							0.60*	16
ENRAGE	−0.54**	28			0.55*	15	0.59*	16
CD40							0.58*	16
CCL25	−0.59**	28					0.51*	16
TWEAK							0.53*	16
STAMBP							0.50*	16

There were no correlations of 0.5 or more in MOM week 1 and infant plasma week 1.

*Rho* Correlation coefficient Spearman’s rho.

*p  < 0.05, **p < 0.01, ^Ɨ^*p* < 0.001.

### Infant sepsis and inflammatory proteins in plasma

All infant plasma proteins were compared on group level depending on sepsis or no sepsis (Supplementary Table 3). Group comparisons between infants with sepsis versus no sepsis demonstrated significantly different inflammatory proteins at weeks 4 and 12, illustrated in Fig. 3e. At week 1, the only protein with significant differences in plasma between infants who developed sepsis and infants who did not was MMP10, with higher levels in infants with sepsis (Supplementary Table 3).

## Discussion

In this study, we demonstrate for the first time an association between antisecretory factor in mothers’ own milk following preterm birth and the development of sepsis in the preterm born infant. This holds true even after adjusting for the total intake of mothers’ milk, suggesting that the finding not only reflects a general positive effect of breastmilk, but that early AF enriched nutrition is in fact beneficial for preterm infants. We also find a correlation between higher AF levels in mothers’ milk over time and less adverse outcomes for the infant, suggesting that AF may contribute to the protective effects of breastmilk regarding infections and inflammatory morbidities of prematurity. This is supported by our finding that low levels of AF in mothers’ own milk in the first week after birth is associated with more inflammatory proteins in the infant’s plasma at four weeks of age, particularly Interleukin-8, which is also linked to sepsis.

Our results are in line with recent studies that have demonstrated a higher degree of inflammation in preterm infants compared with term infants,^3,44^ and inflammation, as well as sepsis, is known to contribute to morbidity, complications and adverse outcomes in infants born preterm.^8,45^

The potential benefit for the infant of a higher level of AF-compleasome in MOM will depend on the amount of MOM the infant receives. To adjust for differences in enteral intake of MOM we calculated a “concentration” of total AF-compleasome intake related to total nutrition and were able to show that a higher AF MOM intake in week 1 was associated with less sepsis. Hence, a high level of AF-compleasome in MOM during the first postnatal week, as well as a high AF MOM intake, may be a part of the anti-inflammatory properties of breastmilk, helping to protect infants from infections and inflammatory morbidities. In other clinical settings, AF is effective in ameliorating inflammatory conditions. In a randomized controlled trial where active AF was given to children with diarrheal diseases, earlier recovery was demonstrated in the AF-treated group compared to the control group.^39^ Furthermore, results from studies using food for specific medical purposes containing active AF demonstrate a significant reduction in intracranial pressure after severe head trauma in adults.^46,47^ Similarly, to the active, preformed AF given in these studies, high AF-compleasome levels in MOM may be involved in the anti-inflammatory protection of the infant.

Gestational age showed no association with AF-compleasome levels in MOM in our material. However, infants with sepsis had lower gestational age. They attained full enteral nutrition later than infants who did not develop sepsis, leading to longer time with intravenous lines and less intake of MOM and, subsequently, less intake of AF. An early start of enteral nutrition may promote the transition from parenteral to enteral nutrition and improve the maturation of the gastro-enteral system and feeding tolerance.^48^ Parenteral nutrition for a longer period is associated with a higher risk for infection and sepsis in preterm infants, related to an increased risk for infection by the need for intravenous lines.^49^ Parenteral nutrition will inevitably lead to less intake of AF via MOM, which must be considered when interpreting the present results.

To assess the inflammatory status of the infant, we analysed a large panel of proteins to screen for inflammatory markers in infant plasma. In the early period, weeks 1 and 4 after birth, an inverse correlation between several marker proteins and AF-compleasome in MOM was determined, indicating higher levels of AF-compleasome in MOM to be associated with a lower degree of inflammation in the infant. A correlation between higher AF-compleasome in MOM week 1 with lower levels of IL-8 was likewise determined in infant plasma week 4. The potential link between the anti-inflammatory effects of AF in MOM and the neonatal outcome is supported by higher levels of IL-8 in infant plasma week 4 determined in infants developing sepsis. The biomarker IL-8 has previously been described to be higher in infants with proven late-onset sepsis.^50^ A sustained inflammatory condition in preterm infants has been associated with elevated levels of IL-1, IL-6, IL-8, and TNFα.^45^ Our results support this finding as infants who developed sepsis demonstrated higher levels of not only IL8, but also TGFα, HGF, TNFSF14 and CCL20 in plasma week 4. These markers of inflammation, including IL-6, were inversely correlated to the level of AF-compleasome in MOM, further strengthening the potential anti-inflammatory role of AF. In adults, higher plasma levels of HGF in patients with sepsis, has been associated with a poorer prognosis.^51^

Infants who developed sepsis had higher levels of the proteins CCL20, CCL23 and CCL25 in infant plasma week 4 compared to infants who did not. CCL25 is a chemokine highly expressed in the thymus and the small intestine and has been suggested to be an important chemokine in mucosal immunity.^52^ Increased levels of CCL25 have been suggested to be involved in the pathogenesis of inflammatory bowel diseases (IBD) and correlated with inflammatory activity.^53^ A larger thymus size has been described as associated with breastfeeding^54,55^ and intake of donor human milk.^56^ Higher levels of CCL20 and CCL23 has also recently been described in infants with sepsis.^57^ Our results are in line with that and a higher level of AF-compleasome in MOM week 1 was found to be counteractive. The association between higher AF and lower levels of CCL20, CCL23 and CCL25 in infant plasma, and lower plasma levels in infants who did not develop sepsis support our hypothesis that AF may have protective properties.

Interestingly, we notice a shift in the later period at week 12 when higher AF-compleasome in MOM were instead associated with higher levels of several of the inflammatory proteins in infant plasma, for example, TNFSF14 and LAPTGFB1, in contrast to their inverse relation to AF at week 4. Furthermore, infants who had developed sepsis had lower levels of TNFSF and LAPTGFB1 in plasma at week 12. LAPTGFB1 is an important component of extracellular matrix and form latent complexes with Transforming Growth Factor Beta (TGFB) and are involved in the regulation of TGFB activity.^58^ In adults, TGF-beta 1 is important for the development and maturation of immune cells, to maintain immune tolerance, homeostasis, and regulation of immune responses. Deviations in control of TGF-beta 1 actions, caused by for example infections, can tip the balance from regulated physiological to excessive pathological repair and thereby impair organ function.^59^

Previously reported by others, reactivating mothers who were IgG seropositive for human cytomegalovirus (HCMV) demonstrated higher TNFSF14, MCP2, and IL-8 in breast milk in early lactation.^60^ A recent study explored the effect of treatment with the active peptide of AF, AF-16, in glioma-bearing mice, human and murine cells and demonstrated prolonged survival, decreased tumour size, changes in macrophage and T-cells invasion, and increased pro-inflammatory markers.^61^ They found modulation of several inflammatory markers, for example, IL-8, MCP2, TNFSF14, by AF-16,^61^ which may strengthen the associations demonstrated in the present study with a suggested role of AF in regulation of inflammatory processes. However, the mechanisms of positive or inverse associations need to be further studied.

Dramatic changes in protein levels in peripheral blood in preterm infants have been described during the first week of life.^44^ Proteins with increasing levels are commonly of liver origin, as proteins involved in complement and coagulation, for example, C3d, suggesting a rapid activation of the immune response.^44^ AF has also been described as involved in regulating the complement system,^32^ which is important for the innate immune system. Thus, in the acute phase of herpes simplex encephalitis infection (HSE), high levels of AF-compleasome in cerebrospinal fluid in adult patients have been associated with a better long-term outcome.^62^ In rats, upregulation of endogenous AF has been demonstrated to enhance hippocampal long-term potentiation (LTP) mediated through disinhibition of CA1pyramidal neurons, leading to enhanced spatial learning and short-term memory.^63^

There is a knowledge gap regarding the effect of preterm infant nutrition on the immune system, and clinical studies identifying changes in the immune system related to various nutrition strategies are needed.^64^ Our results of higher AF-compleasome levels in MOM and AF MOM intake associated with less sepsis add information in this field.^64^ Early human milk, colostrum, is known to be high in bioactive and immunological factors,^65^ with decreasing levels of most factors over time and a suggested role in the early programming of the infant immune system with effects on later health and disease.^66,67^ Early oropharyngeal administrated colostrum has been shown to reduce the inflammatory response in preterm infants.^68^ We speculate that our findings of higher AF-compleasome levels in MOM in colostrum, with decreasing levels over time, and the association between higher AF-compleasome levels in MOM week 1 with less sepsis in preterm infants, suggest AF to be of importance during the first days after birth.

In the present study, there was an interindividual variance in AF-compleasome levels in MOM between different mothers. An interindividual variation of AF-compleasome has also been shown in cerebral fluid in HSE patients in samples collected in the acute phase of the disease,^62^ where the time factor, as well as possible genetic factors, are discussed. Except for higher levels in MOM from mothers of multiples, this variance could not be explained by maternal or delivery characteristics. Further studies are needed to conclusively determine factors associated with maternal AF-compleasome levels in MOM. Unfortunately, we did not have information on maternal diet or HCMV status.

Previous studies on AF in animals have demonstrated that levels of active AF in plasma and milk can be enhanced through an AF-inducing diet with a protective effect in the offspring related to growth and health.^69^ Our finding that higher AF-compleasome levels in MOM week 1 is associated with a lower frequency of sepsis indicates that interventions to induce increased levels of maternal AF in MOM may be of interest. Identify modifiable factors to decrease the burden of sepsis and other adverse outcomes in preterm infants is important to increase health and quality of life for infants and families, as well as reduce societal costs.^70^

There are several limitations of our study that needs to be considered. The study design does not allow us to draw conclusions on causality and the detected associations to AF in MOM are therefore only suggestive of a protective effect against infection and inflammation. The small sample size and the low numbers in some sub-analyses make it difficult to draw firm conclusions. We have not corrected for multiple analyses in the calculations with the inflammatory protein panel, hence, the results need to be interpreted with caution. The power of the study is limited due to the small sample size, further corrections may have decreased the power. We present all analyses in Supplementary Table 2 for full clarity. Despite limitations, the study is explorative hypotheses-generating for future larger studies.

## Conclusion

Mothers’ own milk with naturally high amounts of antisecretory factor during the first week following preterm birth, is associated with lower risk for the infant to later develop sepsis and show signs of inflammation in plasma, particularly measured as lower Interleukin-8. The results suggest that AF-compleasome in MOM may be one important component in the anti-inflammatory and protective effect of breastmilk for preterm-born infants and a possible target for future interventions.

### Supplementary information

Supplementary Table 1

Supplementary Table 2

Supplementary Table 3

## Data Availability

The datasets generated during and/or analysed during the current study are available from the corresponding author on reasonable request.
