# Supplementary material for: Antisecretory factor in breastmilk is associated with reduced incidence of sepsis in preterm infants
Source: Pediatr Res. 2023 Nov 24;95(3):762–9. doi: 10.1038/s41390-023-02909-3 (PMC10899102; doi:10.1038/s41390-023-02909-3)
Supplement: Supplementary file 1 — Supplementary Table 1 [file 41390_2023_2909_MOESM1_ESM.pdf]

Supplementary table 1. The plasma protein panel (ProSeek)

Abbreviation	Protein
ADA	Adenosine Deaminase
ARTN	Artemin*
AXIN1	Axin-1
BetaNGF	Beta-nerve growth factor*
CCL19	C-C motif chemokine 19
CCL20	C-C motif chemokine 20
CCL23	C-C motif chemokine 23
CCL25	C-C motif chemokine 25
CCL28	C-C motif chemokine 28 *
CCL3	C-C motif chemokine 3
CCL4	C-C motif chemokine 4
CXCL1	C-X-C motif chemokine 1**
CXCL10	C-X-C motif chemokine 10
CXCL11	C-X-C motif chemokine 11
CXCL5	C-X-C motif chemokine 5
CXCL6	C-X-C motif chemokine 6
CXCL9	C-X-C motif chemokine 9
CASP8	Caspase-8
CD40	CD40L receptor
CDCP1	CUB domain-containing protein 1
CST5	Cystatin D
DNER	Delta and Notch-like epidermal growth factor-related receptor
CCL11	C-X-C motif chemokine 11
X4EBP1	Eukaryotic translation initiation factor 4E-binding protein 1
FGF19	Fibroblast growth factor 19
FGF21	Fibroblast growth factor 21
FGF23	Fibroblast growth factor 23
FGF5	Fibroblast growth factor 5*
Flt3L	Fms-related tyrosine kinase 3 ligand
CX3CL1	Fractalkine
GDNF	Glial cell line-derived neurotrophic factor*
HGF	Hepatocyte growth factor
IFNg	Interferon gamma*
IL1A	Interleukin-1 alpha*
IL10	Interleukin-10
IL10RA	Interleukin-10 receptor subunit alpha*
IL10RB	Interleukin-10 receptor subunit beta
IL12B	Interleukin-12 subunit beta
IL13	Interleukin-13*
IL15RA	Interleukin-15 receptor subunit alpha*
IL17A	Interleukin-17A*
IL17C	Interleukin-17C*
IL18	Interleukin-18**

IL18R1	Interleukin-18 receptor 1
IL2	Interleukin-2*
IL2RB	Interleukin-2 receptor subunit beta*
IL20	Interleukin-20*
IL20RA	Interleukin-20 receptor subunit alpha*
IL22RA1	Interleukin-22 receptor subunit alpha-1*
IL24	Interleukin-24*
IL33	Interleukin-33*
IL4	Interleukin-4*
IL5	Interleukin-5*
IL6	Interleukin-6
IL7	Interleukin-7*
IL8	Interleukin-8
LAPTGFB1	Latency-associated peptide transforming growth factor beta-1
LIF	Leukemia inhibitory factor*
LIFR	Leukemia inhibitory factor receptor
CSF1	Macrophage colony-stimulating factor 1
MMP1	Matrix metalloproteinase 1
MMP10	Matrix metalloproteinase-10
MCP1	Monocyte chemotactic protein 1**
MCP2	Monocyte chemotactic protein 2
MCP3	Monocyte chemotactic protein 3*
MCP4	Monocyte chemotactic protein 4
CD244	Natural killer cell receptor 2B4
NT3	Neurotrophin-3*
NRTN	Neurturin*
OSM	Oncostatin-M
OPG	Osteoprotegerin
PDL1	Programmed cell death 1 ligand 1*
ENRAGE	Protein S100-A12
SLAMF1	Signaling lymphocytic activation molecule*
SIRT2	SIR2-like protein 2
STAMBP	STAM-binding protein
SCF	Stem cell factor**
ST1A1	Sulfotransferase 1A1*
CD6	T cell surface glycoprotein CD6 isoform
CD5	T-cell surface glycoprotein CD5
CD8A	T-cell surface glycoprotein CD8 alpha chain**
TSLP	Thymic stromal lymphopoietin*
TNFb	TNF-beta
TRANCE	TNF-related activation-induced cytokine
TRAIL	TNF-related apoptosis-inducing ligand
TGFa	Transforming growth factor alpha
TWEAK	Tumor necrosis factor (Ligand) superfamily, member 12

TNF	Tumor necrosis factor*
TNFSF14	Tumor necrosis factor ligand superfamily member 14
TNFRSF9	Tumor necrosis factor receptor superfamily member 9**
uPA	Urokinase-type plasminogen activator**
VEGFA	Vascular endothelial growth factor A

** Excluded due to >20% of samples below limit of detection (LOD)*

*** Excluded due to low number of samples analyzed*
