# Supplementary material for: Antisecretory factor in breastmilk is associated with reduced incidence of sepsis in preterm infants
Source: Pediatr Res. 2023 Nov 24;95(3):762–9. doi: 10.1038/s41390-023-02909-3 (PMC10899102; doi:10.1038/s41390-023-02909-3)
Supplement: Supplementary file 2 — Supplementary Table 2 [file 41390_2023_2909_MOESM2_ESM.pdf]

Supplementary table 2. Correlations between antisecretory factor-compleasome in mothers own milk (AF MOM) and infant plasma proteins (PI)

	AF MOM wk 1- Infant plasma wk 1		AF MOM wk 1- Infant plasma wk 4		AF MOM wk 1- Infant plasma wk 12		AF MOM wk 4- Infant plasma wk 4		AF MOM wk 4- Infant plasma wk 12		AF MOM wk 12- Infant plasma wk 12	
Protein	rho	N	rho	N	rho	N	rho	N	rho	N	rho	N
IL8	-.23	33	-.74ł	28	.04	33	-.75*	11	-.11	15	.45	16
VEGFA	-.32	33	-.54**	28	.18	33	-.68*	11	-.03	15	.40	16
CDCP1	-.37*	33	-.46*	28	.28	33	-.39	11	.59*	15	.78**	16
CD244	-.23	33	-.17	19	.42*	25	-.31	11	.27	11	.67**	16
OPG	-.35	24	-.71**	28	.21	33	-.71	7	.18	15	.47	12
LAPTGFB1	-.16	33	-.42*	24	.44*	29	-.66*	11	.18	12	.19	16
IL6	-.16	30	-.45*	28	.17	33	-.79*	8	-.17	15	.59*	13
CXCL11	.04	33	-.37	28	.36*	33	-.26	11	.00	15	.33	16
AXIN1	-.22	33	-.26	28	.19	33	-.49	11	.19	15	.56*	16
TRAIL	-.29	33	-.34	28	.28	33	-.19	11	.23	15	.60*	16
CXCL9	-.34	33	-.47*	28	.46**	33	-.39	11	.02	15	.55*	16
CST5	-.25	33	-.27	28	.08	33	-.52	11	.11	15	.41	16
OSM	-.23	33	-.56**	28	.14	33	-.65*	11	.32	15	.33	16
CCL4	-.15	33	-.50**	28	.18	33	-.67*	11	.16	15	.47	16
CD6	-.18	33	-.06	28	.28	33	-.32	11	.43	15	.83ł	16
TGFA	-.28	33	-.57**	28	.09	33	-.71*	11	-.11	15	.30	16
MCP4	-.02	33	-.34	28	.12	33	-.29	11	-.03	15	.51*	16
CCL11	-.13	33	-.35	28	.14	33	-.46	11	.21	15	.50*	16
TNFSF14	-.31	33	-.62**	23	.40*	29	-.80**	11	.30	14	.68**	16
FGF23	-.23	27	-.51*	28	.09	33	-.56	10	-.04	15	.12	15
MMP1	-.22	33	-.24	28	.13	33	-.38	11	.00	15	.34	16
LIFR	-.26	33	-.39*	24	.10	29	-.49	11	.16	12	.35	16
FGF21	-.31	30	-.13	28	-.04	33	-.36	8	.46	15	.61*	13
CCL19	-.32	33	-.33	28	.35*	33	-.51	11	.27	15	.49	16
IL15RA	-.37*	33	-.28	28	.20	33	-.58	11	.23	15	.59*	16
IL10RB	-.25	33	-.24	28	.04	33	-.60	11	-.01	15	.27	16
IL18R1	-.23	33	-.39*	28	.19	33	-.49	11	.01	15	.39	16
CXCL5	-.14	33	-.34	28	.43*	33	-.33	11	-.06	15	.13	16
TRANCE	-.16	33	.13	28	.21	33	.11	11	.13	15	.16	16
HGF	-.30	33	-.71ł	28	.12	33	-.78**	11	.08	15	.35	16
IL12B	-.17	33	.07	28	.27	33	-.20	11	.06	15	.40	16
MMP10	-.44*	33	-.40*	28	.00	33	-.36	11	-.16	15	.19	16
IL10	-.30	33	-.40*	28	.36*	33	-.57	11	.16	15	.49	16
CCL23	-.26	33	-.61**	28	.14	33	-.61	11	-.05	15	.50	16
CD5	.04	33	.20	28	.43	33	.00	11	.52	15	.57*	16
CCL3	-.19	33	-.46*	28	.26	33	-.57	11	.02	15	.64**	16
Flt3L	-.28	33	-.23	28	.14	33	-.52	11	.11	15	.39	16
CXCL6	-.12	33	-.63ł	28	.49**	33	-.45	11	-.01	15	.29	16
CXCL10	-.16	33	-.35	28	.33	33	-.47	11	-.05	15	.60*	16
X4EBP1	-.26	33	-.35	28	.18	33	-.44	11	.07	15	.33	16
SIRT2	-.31	33	-.41*	28	.25	33	-.56	11	.02	15	.40	16
DNER	-.12	33	-.40*	28	.14	33	-.42	11	.14	15	.27	16
ENRAGE	-.09	33	-.54**	28	.07	33	-.25	11	.55*	15	.59*	16
CD40	-.28	33	-.35	28	.19	33	-.73	11	.05	15	.58*	16
FGF19	-.20	33	-.21	28	-.02	33	-.21	11	.20	15	.40	16
MCP2	-.18	33	-.37	28	.25	33	-.83	11	-.08	15	.48	16
CASP8	.00	33	-.10	28	.09	33	-.36	11	.35	15	.06	16
CCL25	-.26	33	-.59**	28	.31	33	-.39	11	.29	15	.51*	16
CX3CL1	-.27	33	-.42*	28	.25	33	-.51	11	.10	15	.28	16
TWEAK	-.14	33	-.21	28	.19	33	-.43	11	.10	15	.53*	16
CCL20	-.39*	33	-.34	28	.14	33	-.64	11	-.02	15	.45	16
STAMBP	-.25	33	-.28	28	.30	33	-.39	11	.23	15	.50*	16
ADA	-.16	33	-.23	28	.15	33	-.23	11	.10	15	.43	16
TNFB	-.22	33	.06	28	.38*	33	.00	11	.08	15	.30	16
CSF1	-.19	33	-.30	28	.19	33	-.51	11	.04	15	.48	16

*= p <0.05

**= p < 0.01

ł= p < 0.001

Rho = Correlation coefficient Spearman's rho

wk = week
