# Supplementary material for: Antisecretory factor in breastmilk is associated with reduced incidence of sepsis in preterm infants
Source: Pediatr Res. 2023 Nov 24;95(3):762–9. doi: 10.1038/s41390-023-02909-3 (PMC10899102; doi:10.1038/s41390-023-02909-3)
Supplement: Supplementary file 3 — Supplementary Table 3 [file 41390_2023_2909_MOESM3_ESM.pdf]

Supplementary table 3. Plasma proteins in infant plasma week 1, 4 and 12 after birth related to sepsis or no sepsis.

	Week 1							Week 4							Week 12						
	Sepsis			No sepsis			*p*	Sepsis			No sepsis			*p*	Sepsis			No sepsis			*p*
Protein	Mdn	IQR	N	Mdn	IQR	N		Mdn	IQR	N	Mdn	IQR	N		Mdn	IQR	N	Mdn	IQR		
IL6	4.43	.95	9	4.11	2.39	29	.450	3.72	2.04	8	2.73	1.81	23	.082	2.59	.62	9	2.81	1.28	28	.412
IL8	8.03	1.83	10	7.88	1.84	32	.170	8.02	.42	9	6.84	1.02	26	.003**	7.02	.98	9	6.58	.76	33	.171
IL15RA	.74	.82	10	.82	1.11	32	.690	1.44	.94	9	.96	.99	26	.239	1.15	1.17	9	1.58	.84	33	.289
IL10RB	6.74	.82	10	6.86	.79	32	.988	7.27	.85	9	6.99	1.01	26	.565	7.41	1.13	9	7.69	.48	33	.695
IL18R1	6.90	1.35	10	6.97	1.11	32	.647	7.90	1.57	9	7.30	1.14	26	.197	7.44	1.41	9	7.91	.98	33	.673
IL12B	3.67	1.38	10	4.31	1.82	32	.400	4.39	2.24	9	4.90	1.54	26	.540	5.66	1.77	9	6.45	1.13	33	.487
IL10	2.53	1.06	10	2.56	2.10	32	.734	3.58	1.47	9	2.91	1.68	26	.382	2.96	1.45	9	3.76	.84	33	.161
VEGFA	10.45	.95	10	10.37	1.26	32	.871	11.15	1.91	9	10.45	1.10	26	.067	10.33	1.26	9	10.66	1.09	33	.763
TGFA	3.06	.51	10	2.76	.83	32	.232	3.01	1.38	9	2.45	.94	26	.023*	2.82	.96	9	2.51	.61	33	.507
HGF	7.63	.94	10	7.58	1.32	32	.871	7.77	.58	9	6.90	1.31	26	.013*	7.36	.96	9	7.36	.67	33	.630
OPG	6.34	.72	7	6.42	1.60	23	.924	6.86	.88	6	6.16	1.47	19	.138	7.09	.78	7	7.10	.53	25	.532
LAPTGFB1	6.74	.81	10	6.97	1.04	32	.488	7.55	.79	9	7.12	.85	26	.516	6.81	.52	9	7.16	.52	33	.049*
TNFSF14	3.48	1.18	10	3.52	1.03	32	.337	4.18	.85	9	3.85	.93	26	.046*	3.35	1.03	9	3.91	.64	33	.028*
TRAIL	7.12	1.36	10	7.24	.93	32	.965	7.60	.75	9	7.56	.83	26	.725	8.14	1.05	9	8.50	.71	33	.070
TWEAK	6.89	.63	10	7.38	1.03	32	.220	7.01	1.60	9	7.57	1.25	26	.643	7.89	1.44	9	8.58	.60	33	.348
TNFB	3.79	1.34	10	3.98	.80	32	.988	3.62	1.04	9	4.31	.92	26	.382	4.86	1.00	9	5.75	.86	33	.092
TRANCE	3.91	2.12	10	4.72	1.42	32	.352	4.52	2.34	9	5.16	1.15	26	.342	6.85	1.34	9	7.04	.73	33	.857
MCP2	6.88	1.07	10	6.84	1.37	32	.605	7.72	1.42	9	7.32	1.09	26	.590	7.41	.95	9	8.03	.96	33	.673
MCP4	7.76	1.54	10	8.24	1.11	32	.941	8.92	1.50	9	8.01	.98	26	.271	8.46	.95	9	8.54	1.44	33	.718
MMP1	8.48	.55	10	8.54	2.32	32	.626	8.95	2.11	9	8.96	1.45	26	.469	7.40	.73	9	7.73	1.24	33	.275
MMP10	9.89	.49	10	9.34	1.02	32	.037*	9.62	.73	9	9.43	.97	26	.210	8.42	.68	9	8.74	1.10	33	.526
CCL3	4.76	.52	10	4.42	1.29	32	.322	4.79	1.17	9	4.20	1.11	26	.138	4.82	.86	9	4.82	.86	33	.487
CCL4	5.92	.81	10	5.96	1.18	32	.585	6.24	1.02	9	6.06	1.12	26	.119	6.20	.54	9	6.44	.69	33	.143
CCL11	5.72	1.09	10	6.11	1.26	32	.337	6.86	.98	9	6.58	1.37	26	.323	7.04	1.18	9	7.09	1.06	33	.952
CCL19	8.79	.59	10	8.53	1.59	32	.384	8.82	1.61	9	8.35	1.13	26	.342	9.40	1.39	9	9.63	1.19	33	.275
CCL20	6.70	2.03	10	6.76	1.81	32	.894	8.90	1.42	9	7.29	1.42	26	.025*	7.54	.66	9	7.37	1.34	33	.608
CCL23	8.98	1.42	10	8.60	1.76	32	.668	9.31	2.42	9	7.55	1.76	26	.038*	8.09	2.00	9	8.88	1.29	33	.928
CCL25	6.48	1.27	10	6.62	1.43	32	.647	7.01	1.15	9	6.22	1.25	26	.020*	6.13	1.08	9	6.57	.52	33	.740
CXCL5	7.74	.92	10	8.52	1.98	32	.384	8.82	1.97	9	8.99	1.48	26	.985	8.40	1.38	9	9.41	1.63	33	.213
CXCL6	6.86	1.29	10	7.12	1.61	32	.605	8.22	1.60	9	7.40	1.12	26	.067	7.87	.43	9	8.14	1.34	33	.348
CXCL9	4.86	2.22	10	4.83	2.56	32	.941	6.18	1.36	9	5.18	1.45	26	.255	5.50	.86	9	6.32	1.11	33	.119
CXCL10	5.00	3.04	10	5.40	2.74	32	.626	6.67	1.72	9	5.84	1.97	26	.288	6.90	1.39	9	7.29	1.87	33	.224
CXCL11	6.19	1.50	10	7.15	1.17	32	.121	7.65	2.34	9	7.05	1.31	26	.197	7.62	.68	9	8.03	2.00	33	.608
CX3CL1	5.34	.65	10	4.65	1.24	32	.101	5.10	1.75	9	4.74	1.01	26	.271	5.19	1.52	9	5.89	1.00	33	.608
CD5	4.92	.98	10	5.48	.77	32	.170	5.02	.93	9	5.73	1.16	26	.067	6.04	.51	9	6.32	.60	33	.065
CD6	3.34	1.17	10	3.76	.92	32	.308	3.47	1.24	9	4.37	1.28	26	.288	4.78	1.44	9	5.59	1.00	33	.119
CD40	9.76	1.53	10	9.61	1.00	32	.802	10.69	1.14	9	9.76	1.00	26	.061	10.16	.90	9	10.54	.58	33	.695
CD244	4.66	1.60	10	5.16	.80	32	.434	5.41	1.10	9	5.64	.76	26	1.000	5.75	1.10	9	6.41	.43	33	.086
CDCP1	1.84	.85	10	1.96	.99	32	.918	2.31	.66	9	2.23	1.04	26	.469	2.09	.87	9	2.68	.69	33	.224
CSF1	8.64	.44	10	8.59	1.23	32	.894	9.21	1.07	9	8.54	.78	26	.271	8.70	1.07	9	9.41	.96	33	.718
CST5	7.17	.95	10	7.60	1.71	32	.894	8.06	1.42	9	7.91	1.43	26	.160	8.21	1.19	9	7.87	1.23	33	.567
DNER	8.51	.37	10	8.68	.82	32	.469	9.06	1.06	9	8.71	.77	26	.271	8.60	1.03	9	9.25	.77	33	.303
ENRAGE	2.35	.74	10	2.25	1.72	32	.825	3.42	1.86	9	2.34	1.78	26	.056	1.96	1.80	9	1.69	1.08	33	.857
FGF19	7.20	.62	10	6.24	1.80	32	.256	6.74	2.82	9	5.76	1.30	26	.810	6.22	1.38	9	7.33	2.23	33	.065
FGF21	5.39	1.62	9	4.07	1.91	29	.492	6.55	4.05	8	5.92	1.91	23	.842	7.97	2.67	9	8.12	5.00	28	1.000
FGF23	5.33	1.13	8	4.84	3.25	26	.270	4.92	1.39	7	3.90	1.35	22	.055	4.19	.93	7	3.62	1.40	30	.458
Flt3L	7.98	.88	10	8.16	1.30	32	.988	9.25	1.80	9	8.60	1.27	26	.956	9.26	1.68	9	9.24	1.31	33	.630
LIFR	2.59	.91	10	3.05	.93	32	.734	3.25	.80	9	3.02	.90	26	.565	3.07	.94	9	3.61	.73	33	.469
OSM	2.17	1.56	10	2.66	2.20	32	.605	3.02	.56	9	2.12	1.84	26	.086	1.31	2.04	9	1.95	1.31	33	.450
SIRT2	3.73	1.13	10	4.11	1.85	32	.368	3.98	.85	9	3.96	1.51	26	.590	4.09	1.38	9	4.20	.98	33	1.000
STAMBP	5.09	.80	10	5.40	1.39	32	.322	5.64	1.24	9	5.25	1.49	26	.781	4.93	1.35	9	5.41	.82	33	.414
X4EBP1	9.08	.99	10	9.50	1.37	32	.189	8.93	1.01	9	8.82	1.82	26	.897	8.38	1.33	9	8.84	1.29	33	1.000
ADA	5.75	.65	10	5.98	.83	32	.712	5.87	.96	9	5.82	1.41	26	.985	5.76	.42	9	6.06	.60	33	.880
AXIN1	1.75	1.15	10	2.20	1.70	32	.712	2.85	.55	9	2.58	1.12	26	.424	2.76	.41	9	3.00	1.20	33	.526
CASP8	2.11	1.47	10	2.48	1.68	32	.308	2.06	1.10	9	2.22	1.36	26	.926	1.45	1.19	9	1.90	.89	33	.546

Mann-Whitney U-test
Mdn = Median
*= p <0.05
**= p <0.01
